# GnRH Antagonist Improves Pubertal Cyclophosphamide-Induced Long-Term Testicular Injury in Adult Rats

**DOI:** 10.1155/2018/4272575

**Published:** 2018-06-10

**Authors:** Rongrong Xie, Linqi Chen, Haiying Wu, Ting Chen, Fengyun Wang, Xiuli Chen, Hui Sun, Xiaozhong Li

**Affiliations:** ^1^Department of Endocrinology, Children's Hospital of Soochow University, Suzhou 215003, China; ^2^Department of Nephrology, Children's Hospital of Soochow University, Suzhou 215003, China

## Abstract

**Background:**

Gonadal injury following chemotherapy is of increasing importance with the continuous improvement of survival rates. The protection of gonadotropin hormone antagonist (GnRHant) in long-term adult survivors of adolescent cancers and some autoimmune diseases has not yet been evaluated.

**Methods:**

The present study was aimed at longitudinally exploring whether the GnRHant could alleviate testicular damage induced by cyclophosphamide (CPA) in a rat model. Pubertal male rats were assigned to receive CPA with and without GnRHant. CPA was administrated at a single dose (100 mg/kg). GnRHant was started one hour prior to CPA injection and continued for four weeks (0.1 mg/kg, 3 times a week). Body and testes weights, testicular hormones, histological changes, and expression of androgen receptor (AR) in the testis were analyzed when rats matured into adulthood and completed a round of spermatogenesis.

**Results:**

Our results showed that body weight, histological injury, and AR expression in the testis were improved in the GnRHant + CPA group. However, testes weight and testicular hormones (anti-Müllerian hormone, inhibin B, and testosterone) did not markedly change.

**Conclusion:**

Our results indicate that the GnRHant administration before and after CPA in pubertal rats can protect long-term testicular injury induced by CPA via increased AR expression in the testes.

## 1. Introduction

As the long-term survival rates for childhood cancers and some severe autoimmune diseases improve [1–3], the quality of life of these survivors deserves more attention. Gonadal damage is one of the most serious late effects after treatment in adolescence with aforementioned diseases. Cyclophosphamide (CPA) as a cytotoxic alkylating agent is widely used as an anticancer and immunosuppressive agent. Beside acute adverse effects, CPA may cause long-term or permanent gonadal damage on young male patients [4, 5]. Dividing cells are known to be more sensitive to the cytotoxic effects of the alkylating agents than resting cells. Thus, postpubertal testis is one of the target organs for damage effects. It is known that spermatogenesis is affected by gonadotropins and suppression of them can suppress spermatogenesis. Therefore, it is logical to conclude that the inhibition of pituitary-gonadal axis during chemotherapy could protect spermatogenesis.

There are several noninvasive methods for the inhibition of pituitary-gonadal axis including usage of agonists and antagonists of GnRH [6–8]. GnRH agonists have an initial stimulatory effect on the gonads, thus delaying gonad suppression, which is expected to occur after an about two-week period [9]. This waiting time is not acceptable in patients with high-risk cancers or rapidly progressive autoimmune diseases. In contrast, GnRH antagonists cause immediate gonad suppression by competitively blocking GnRH receptors in the pituitary. With their immediate onset of action, GnRH antagonists may be more suitable for use immediately before initiation of chemotherapy. The testicular cytotoxic effect of CPA targets Sertoli cells, Leydig cells, and germ cells [10, 11]. In these cells, androgen receptor (AR) can be detected [12, 13]. And AR is essential for spermatogenesis and male fertility [12, 14]. However, there are few animal studies to date that assessed the role of a GnRH antagonist in future fertility protection during chemotherapy especially for pubertal stage via AR expression.

Thus, the aim of the present study was to longitudinally evaluate whether the administration of a GnRHant prior to the administration of CPA in male pubertal rats could prevent from testicular damage during a long-term follow-up to adulthood.

## 2. Materials and Methods

### 2.1. Animals

24 pubertal male Wistar rats aging 5 weeks were obtained from the Experimental Animal Center, Soochow University (Suzhou, China) [15, 16]. Room temperature and humidity were maintained at 18–22°C and 50–60%, respectively, with 12-hour light-dark cycles. Water and rat food were available ad libitum. All rats were acclimatized for 2 days before the experiment. This study was approved by the Ethics Committee on Animal Experiments at the Children's Hospital of Soochow University.

### 2.2. Experimental Protocols

CPA (Baxter Oncology GmbH, Kantstr, Germany) diluted in 0.9% saline solution, resulting in a 20 mg/ml dose, was administrated to rats at a single dose of 100 mg/kg, only once. The dose of CPA was selected according to previous studies that demonstrated significant damage in sperm parameters and testicular toxicity in rats. Beside these, this dose is equivalent to the therapeutic large dose for humans. GnRHant (Cetrorelix, Merck Serono, France) dissolved in sterilized distilled water was injected at a dose of 0.1 mg/kg 1 hour prior to the CPA injection and continued for 4 weeks (3 times a week). This dose is known to inhibit the luteinizing hormone surge in about 1 hour. Previous studies had shown that the maximum serum concentration of GnRHant is reached after two hours. CPA and GnRHant were injected aseptically via the peritoneum, on the inferior abdominal area next to the right hind leg. The male rats were randomly divided into four groups equally as follows:

- Control group (*n* = 6). The rats received placebo saline 3 times a week, continued for 4 weeks. Placebo sterilized distilled water was administrated 1 hour after the first saline injection, only once.
- CPA group (*n* = 6). The rats received placebo saline 3 times a week, continued for 4 weeks. CPA was administrated 1 hour after the first saline injection, only once.
- GnRHant + CPA group (*n* = 6). The rats received GnRHant 3 times a week, continued for 4 weeks. CPA was administrated 1 hour after the first GnRHant injection, only once.
- GnRHant group (*n* = 6). The rats received GnRHant 3 times a week, continued for 4 weeks. Placebo sterilized distilled water was administrated 1 hour after the first saline injection, only once.

Since rat spermatogenesis from spermatogonia to mature sperm takes about 9 weeks, in our study, all rats were sacrificed under anaesthesia of 3% chloral hydrate (1 ml/kg) 9 weeks after the last GnRHant injection. The blood sample was collected from the left ventricle and the serum was separated and frozen at −20°C. The testes were excised. The right testis was fixed for histological and immunohistochemistry evaluation; the left one was frozen at −80°C until Western blot analysis.

### 2.3. Body and Testicular Weights

The weight of each rat was recorded before killing. Both testes were excised out and weighed immediately following necropsy.

### 2.4. Hormone Measurements

Serum testosterone, anti-Müllerian hormone (AMH), and inhibin B were measured using ELISA according to the manufacturer's instructions of the kits (Biomatik, USA).

### 2.5. Histology

The right testis was fixed in 10% formalin, dehydrated in ethanol, and embedded in paraffin. 10 *μ*m serial sections were stained with hematoxylin-eosin (HE). Images were obtained under the microscope (Olympus AX70, Tokyo, Japan).

### 2.6. Immunohistochemistry Analysis for AR

Testis sections were deparaffinized, dehydrated, blocked, then incubated at 4°C for 24 h with the primary rabbit anti-AR polyclonal antibody (1 : 200, N-20, sc-816, Santa Cruz, CA, USA), followed by reaction with corresponding/HRP-conjugated goat anti-rabbit secondary antibody (074-1506, KPL), then incubated with DAB staining kit. Sections were analyzed and imaged using an Olympus digital camera. Nine fields were chosen randomly for each specimen [17–19]. The expression of AR-positive cells was counted under a microscope. The evaluation of AR immunoexpression was done using Image-Pro Plus software (version 6.0, Media Cybernetics, USA). Signals were quantified by scanning densitometry, and the average optical density (OD) was obtained.

### 2.7. Western Blotting

The expression level of AR in the testis was detected by Western blot. Equal proteins were separated by 10% SDS-PAGE and electrotransferred to polyvinylidene difluoride (PVDF) membranes, which were then incubated with the primary antibodies against AR (1 : 1000, N-20, sc-816, Santa Cruz, USA) and *β*-actin (1 : 1000, Sigma). The membranes were then incubated with HRP-conjugated secondary antibody (1 : 1000, Santa Cruz, sc 20137,USA). AR band intensities were normalized to the endogenous control *β*-actin and measured by using ImageJ 2 (National Institutes of Health, USA).

### 2.8. Statistical Analysis

For comparison of the results, one-way analysis of variance (ANOVA) was performed for parametric data. Statistical analyses were performed on SPSS 18.0. *p* values < 0.05 were considered statistically significant.

## 3. Results

### 3.1. General Condition and Body and Testicular Weight Changes

In CPA and CPA + GnRHant groups, different degrees of hair loss, ungroomed hair, reduced activities, and decreased appetite were observed in the first week after CPA injection. In any group, no abnormal behaviors were noted and none of them died throughout the experiment. Changes of the body and testicular weights were shown in Table 1. The body weight decreased significantly in the CPA group compared with the remaining groups (*p* < 0.001). Improvement of the body weight was seen in GnRHant + CPA group compared with the CPA group. However, testicular weight did not change markedly in any group.

### 3.2. Serum Levels of Testicular Hormones

The serum levels of testosterone, AMH, and inhibin B were not significantly different in any group (Table 2). CPA treatment in the pubertal stage did not change the testicular hormonal levels significantly in comparison with the control group.

### 3.3. Histological Changes

**Figure 1:**
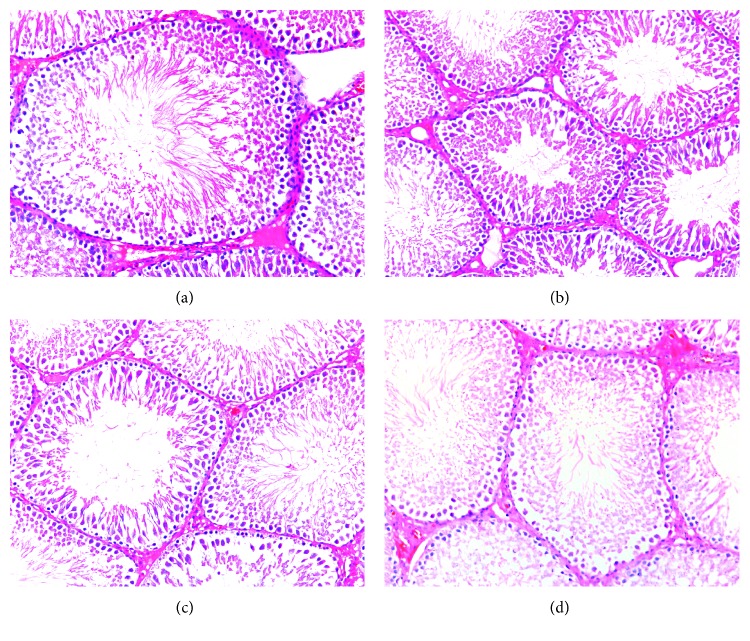
Representative photomicrograph of testis sections. (a, d) From the control groups. Note the regular arrangement of spermatogenic cells and seminiferous tubules. (b) From the CPA group. Note the nuclear pyknosis and cytoplasmic microvacuolization of spermatogenic cells and relatively decreased diameter of seminiferous tubules. (c) From the GnRHant + CPA group. Note the relative recovery of spermatogenic cells and seminiferous tubules. HE staining, ×200.

In the control and GnRHant groups (Figures 1(a) and 1(d)), the seminiferous tubules had a normal, thick, and smooth germinal epithelium. Thus, the group that received only GnRHant did not differ from the control group. The regular seminiferous tubules and their lumen contained abundant spermatozoa, and interstitial spaces contained obvious Leydig cells under light microscopy.

In the CPA group (Figure 1(b)) compared with the control group, nuclear pyknosis and cytoplasmic microvacuolization were present in the spermatogenic cells. Irregular seminiferous tubules and relatively decreased diameter of seminiferous tubules could be observed. Sperm cells in the lumina of seminiferous tubules were decreased relatively. However, when compared with the CPA group, the GnRHant + CPA group (Figure 1(c)) showed less prominent changes in the histology of seminiferous tubules and spermatogenic cells. The morphology of germinal epithelium was almost similar with the control group.

### 3.4. Distribution and Expression of AR Protein in the Testis

**Figure 2:**
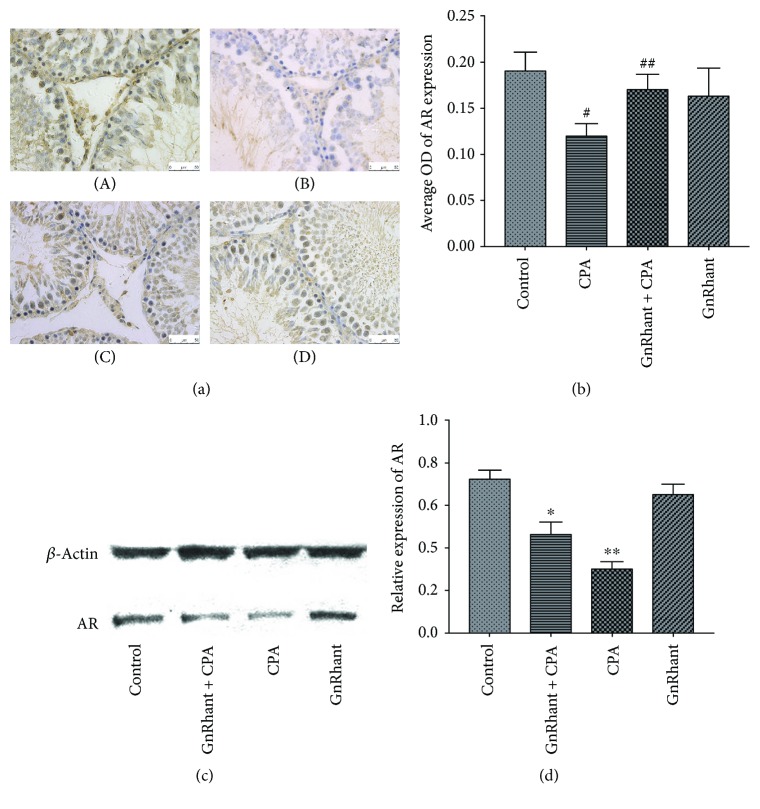
Representative photomicrograph of AR immunochemistry and Western blot assays in the testis. (a) (A) The control group showed strong AR immunostaining in the Sertoli, Leydig, and peritubular myoid cells. (B) The CPA group showed markedly decreased AR expression. (C) The GnRHant + CPA group showed relatively restored AR expression. (D) The GnRHant group showed the same strong AR expression as the control group. (A, B, C, and D ×400). (b) Quantitative analysis of AR expression by immunohistochemistry in the testis. ^#^Significantly different from the other three groups (*p* < 0.001). ^##^Much higher than that in the CPA group (*p* < 0.001). (c) Representative photographs of Western blot for AR protein expression. (d) Quantitative analysis of AR expression by Western blot in the testis. ^∗^*p* < 0.001 compared with the other three groups (*p* < 0.001). ^∗∗^*p* = 0.03, compared with the GnRHant + CPA group.

To analyze the distribution and expression of AR in the testis with and without GnRHant, immunohistochemistry and Western blot assays were performed. The presence of AR immunostaining in the testis was detected in the Sertoli cells, Leydig cells, and peritubular myoid cells (Figure 2(a)). The number of AR-positive cells was markedly decreased in the CPA group (Figure 2(a), B), in comparison with other groups (*p* < 0.05). Significant improvement was noted in the GnRHant + CPA (Figure 2(a), C), as compared with the CPA group (*p* < 0.05). As shown in Figure 2(b), with the qualitative analysis of AR immunohistochemistry by calculating average optical density (OD), the CPA group decreased significantly in comparison with the control group (*p* < 0.001). The AR level of the GnRHant + CPA group was much higher than that of the CPA group (*p* < 0.001) but had no difference with the control or GnRHant groups (*p* > 0.05). As shown in Figures 2 and 2, the CPA group of the AR protein expression detected by Western blot was lower than the other three groups (*p* < 0.001). The AR protein level of the GnRHant + CPA group was higher than that of the CPA group (*p* = 0.03).

## 4. Discussion

Protection against the adverse effects of CPA on pubertal testicular function to improve the quality of their adult life is the challenge for clinicians and researchers. Evidence suggests that prepubertal patients are less sensitive to the adverse effects of the chemotherapy [20, 21]. Thus, medications that are able to make the gonads quiescent and less sensitive to chemotherapy-induced cytotoxicity could protect fertility.

Delaying cancer and autoimmune disease treatment can pose serious risks to the patients. Therefore, a drug that can promote immediate testicular suppression is preferable. Because of the immediate suppression of the action of GnRH antagonists, their use would eliminate the 2- or 3-week waiting period, which is usually required to suppress testicular activity when using GnRH agonists [22, 23].

In this study, CPA administration led to a significant decrease in adult weight especially in the CPA group. We noted that the rats in the two CPA-administrated groups had reduced activities and decreased appetite in the first week after CPA injection. It was concluded that without any protective measures, the weight loss was severe in the rats treated with only once high dose of CPA (100 mg/kg), which is equivalent to the therapeutic large dose (16 mg/kg) for humans. We have not observed significant changes in the testicular weight and hormones in any treatment group. Other's investigations showed that the testicular weight and hormones decreased significantly soon after chemotherapy without 9-week cycles of sperm maturation [24–26]. However, we evaluated the testicular function until adulthood after CPA administration. Our study was designed to longitudinally evaluate the potential testicular function in adult rats, while treated in the pubertal stage. This maybe the difference in terms of testicular weight and hormones. However, inhibin B can be a marker of germ cell function; the levels of inhibin B or the ratio of inhibin B/FSH do not represent normal spermatogenesis in patients who have undergone cancer treatment during childhood [27].

In our study, a histological impairment in spermatogenesis could be seen in the group that received only CPA. The testis sections revealed several histological changes such as nuclear pyknosis and cytoplasmic microvacuolization in the spermatogenic cells, irregular seminiferous tubules, and relative loss of the spermatogenic cells in the CPA-treated rats. It indicated that even just one single dose of CPA (equivalent to the large dose for humans) administrated during puberty could cause testicular long-term impairment till the adult stage. Mohammadi et al. have reported that after 6 weeks, histological damage could be detected after a single dose of CPA 100 mg/kg injection in adult male rats [24]. Our results have shown that when GnRHant was administrated before and after CPA injection, it could partially improve the histological injuries induced by CPA in the testes.

The biological actions of physiological androgens are mediated by the AR. AR plays critical roles in spermatogenesis and male fertility [28, 29]. The absence of AR will cause arrest of spermatogenesis [14, 30]. The level of AR is essential for normal structure and function of male reproductive system. In our study, we used immunochemistry and Western blot assays to analyze the expression of AR protein and observed that the number of AR-positive cells and the level of AR protein were markedly reduced in the CPA group rats compared with the control group. It indicated that CPA could reduce AR expression in the testes. Reduced AR has an influence on the functions of Sertoli cells in supporting and nurturing germ cells, causing the arrest of spermatogenesis at the diplotene spermatocyte stage of meiosis [13, 31]. Reduced AR in the Leydig cells mainly affects steroidogenic functions. If AR expression is reduced, spermatogenesis at the round spermatid stage is arrested [30]. According to our results, there is no correlation between AR expression and serum testosterone level. This outcome is possibly due to either the rats we started to treat are in the pubertal stage not in the adult stage or our long-term observation period is from puberty to adulthood. There may be no obvious difference as for the levels of testosterone. Further study is necessary to elucidate this difference. Reduced AR in peritubular myoid cells results in decreased sperm output. Therefore, it can be concluded that the decreasing of AR expression after CPA administration is probably directly linked to impairment of spermatogenesis. Our results showed that the administration of GnRHant prior to CPA could effectively increase AR expression in the testes compared with the CPA group and protect fertility.

However, there were some limitations in this study. First, AR expression in the Sertoli cells changes dramatically in the cycle of the seminiferous epithelium in the adult male rat. We should compare the immunostaining sections at the same stage in our detailed future work. Second, we analyzed the body and testes weights, testicular hormones, and histomorphological and immunohistochemical changes in the testis when rats matured into adulthood. We should add sperm quality analysis that is sensitive to evaluate the male reproduction in future studies. Third, we would add spermatogonia count for better analysis. As for empty spaces in the seminiferous tubules in slides of four groups, they may be artificial due to embedding processing of the tissues. We should improve this situation in our future work.

## 5. Conclusion

The present study indicates that the administration of CPA in pubertal male rats has a long-term testicular damage till adulthood. And the administration of GnRHant by suppressing the hypothalamic gonadal axis has a partial protection on the future adult testicular function of rats undergoing pubertal chemotherapy and immunosuppressive treatment with CPA. This study provides the evidence that GnRHant can be used as an adjuvant therapy to prevent the pubertal testes impaired by CPA in clinical practice and then improve the quality of adult life.

## Figures and Tables

**Table 1:** Body and testes weights.

Parameters	Control	CPA	GnRHant + CPA	GnRHant
Body wt (g)	304.67 ± 15.44	259.33 ± 14.62^∗^	307.50 ± 12.47	321.00 ± 18.43
Testes wt (g)^a^	2.68 ± 0.13	2.65 ± 0.31	2.67 ± 0.25	2.80 ± 0.09

^a^The testes weight is the total weight of the right and left testes. ^∗^Significantly different from the other three groups (*p* < 0.001).

**Table 2:** Serum levels of testicular hormones.

Parameters	Control	CPA	GnRHant + CPA	GnRHant
Testosterone (ng/ml)	5.96 ± 0.39	6.13 ± 0.45	5.75 ± 0.39	6.13 ± 0.44
AMH (ng/ml)	32.39 ± 3.41	33.69 ± 2.30	30.99 ± 4.98	32.73 ± 2.44
Inhibin B (pg/ml)	84.78 ± 5.64	79.69 ± 11.96	84.70 ± 10.94	85.86 ± 8.90

## Data Availability

The data can be obtained by contacting the corresponding author.
